# Lipid management in type 2 diabetes and non-HDL-cholesterol: target all atherogenic lipoproteins

**DOI:** 10.1186/s12933-026-03166-4

**Published:** 2026-04-12

**Authors:** Julia Brandts, Marlo Verket, Alberto Zambon, Nikolaus Marx, Dirk Müller-Wieland, Massimo Federici

**Affiliations:** 1 Department of Internal Medicine I—Cardiology, RWTH Aachen University, Aachen, Germany; 2 Department of Public Health and Primary Care, Imperial College London, London, UK; 3 Department of Medicine - DIMED, University of Padua, Padua, Italy; 4 Department of Systems Medicine, University “Tor Vergata” of Rome, Rome, Italy; 5 IRCCS NEUROMED, Pozzilli, Italy

**Keywords:** Diabetes, Hypercholesterolaemia, Triglycerides, Non-HDL cholesterol

## Abstract

Atherosclerotic cardiovascular disease (ASCVD) remains a leading cause of morbidity and mortality in individuals with diabetes, partly driven by dyslipidemia. While low-density lipoprotein cholesterol (LDL-C) reduction is the primary target of lipid management, many patients with diabetes exhibit mixed dyslipidemia characterised by elevated triglycerides and increased concentrations of atherogenic remnant lipoproteins, which are more comprehensively captured by non-high-density lipoprotein cholesterol (non-HDL-C). Current guidelines from international societies, including the American Diabetes Association (ADA), the American Association of Clinical Endocrinology (AACE), and the European Society of Cardiology (ESC), recommend LDL-C and non-HDL-C targets based on individual cardiovascular risk profiles. Despite clear therapeutic algorithms, lipid target attainment remains suboptimal in routine clinical practice, necessitating more intensive and individualised treatment strategies. Lipid-lowering therapies, including statins, ezetimibe, bempedoic acid and PCSK9 inhibitors, effectively reduce LDL-C and non-HDL-C, significantly lowering cardiovascular risk. Triglyceride-lowering therapies, including omega-3 fatty acids and fibrates, have demonstrated substantial reductions in triglyceride levels, but their impact on cardiovascular outcomes remains uncertain. Given the heterogeneity of dyslipidemia in diabetes, non-HDL-C and apolipoprotein B (apoB) have emerged as superior markers for assessing atherogenic burden. While LDL-C reduction remains central, additional efforts are needed to optimise the management of residual atherogenic lipoprotein particles in diabetes. Future research should focus on refining risk stratification, improving lipid target attainment, and integrating novel lipid-modifying agents to enhance cardiovascular outcomes in this high-risk population.

**Research insights**

**What is currently known about this topic?**

Individuals with type 2 diabetes have an increased risk of atherosclerotic cardiovascular disease, partly driven by mixed dyslipidemia.

Reduction of LDL-C Is the primary therapeutic target and is strongly associated with cardiovascular risk reduction. Current international guidelines recommend risk-adapted LDL-C goals.

Despite effective lipid-lowering therapies, lipid target attainment remains suboptimal, and residual atherogenic risk persists in many patients with diabetes.

**What is the key research question?**

How can lipid management in individuals with type 2 diabetes be optimized beyond LDL-C reduction to more effectively address residual atherogenic lipoproteins and improve cardiovascular outcomes?

**What is new?**

This study identifies non-HDL-C and apolipoprotein B (apoB) as more comprehensive markers of total atherogenic lipoprotein burden in diabetic dyslipidemia.

**How might this study influence clinical practice?**

It supports a more individualized, multimodal approach to lipid management in diabetes that extends beyond LDL-C alone, incorporating non-HDL-C/apoB, risk stratification tools and selected imaging findings to better address residual cardiovascular risk.

## Introduction

Atherosclerosis is driven by Apolipoprotein B (apoB)100-containing lipoproteins, and their impact is cumulative. Lowering these lipoproteins at any stage reduces risk of cardiovascular (CV) events. A 1 mmol/L (38.67 mg/dL) reduction in low-density lipoprotein cholesterol (LDL-C) lowers CV risk by a fifth, independent of the treatment approach or patient population [1]. Differences in outcomes across lipid-lowering trials, whether involving statins, ezetimibe, or proprotein convertase subtilisin/kexin type 9 (PCSK9) inhibitors, are primarily due to variations in absolute LDL-C reductions and treatment duration.

Therefore, lipid management is central to reducing CV risk in diabetes, given the strong causal link between atherogenic lipoproteins and CV disease (CVD). Individuals with diabetes have at least a 2-fold higher risk of developing CVD compared to those without diabetes [2, 3]. However, in clinical practice, diabetes-associated CV risk is often underestimated, leading to delayed and suboptimal lipid-lowering treatment.

**Fig. 1:**
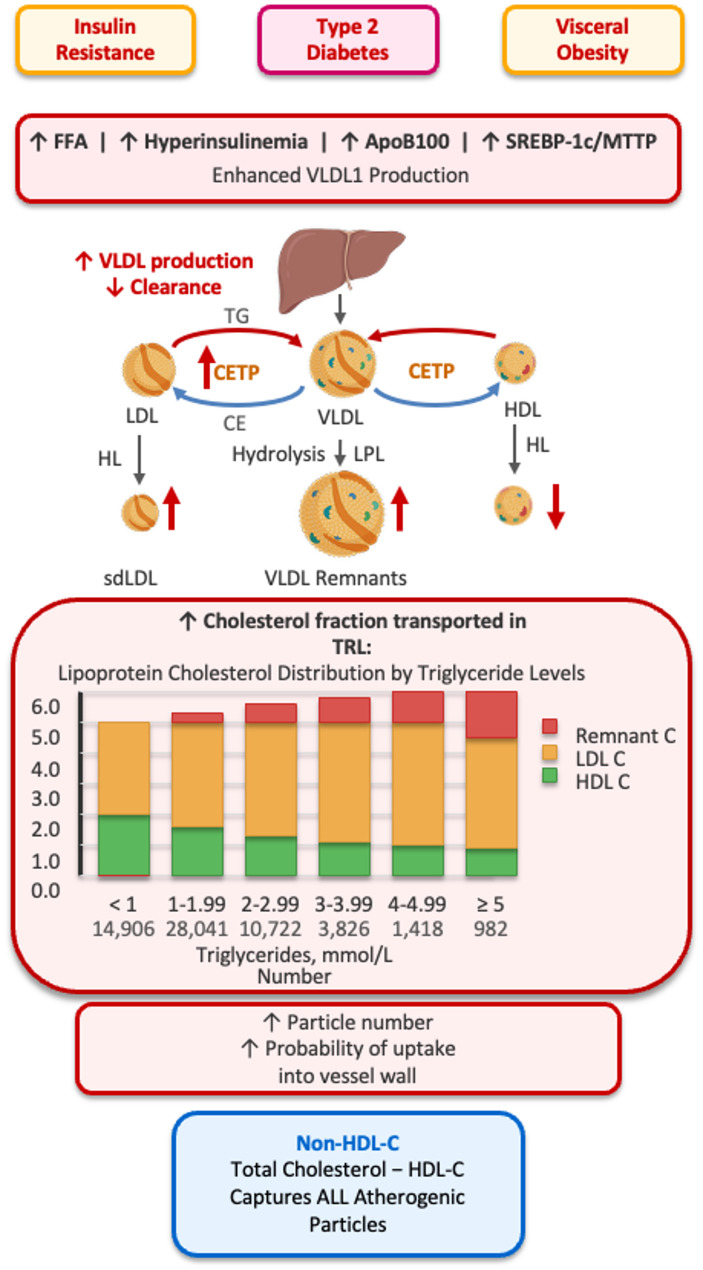
Overview and key aspects of non-HDL-cholesterol in type 2 diabetes

Furthermore, diabetic dyslipidemia often presents as mixed dyslipidemia, where LDL-C alone may not fully reflect atherogenic apoB burden and atherosclerotic cardiovascular disease (ASCVD) risk [4]. This underscores the importance of comprehensive lipid management strategies beyond LDL-C lowering alone (Fig. 1). The following sections will explore treatment targets and goals and therapies for improving CV and clinical outcomes in individuals with type 2 diabetes (T2D).

## Dyslipidemia in insulin resistance and type 2 diabetes mellitus

Dyslipidemias are metabolic disorders with abnormalities in lipid parameters and are classified into two forms: genetic and acquired factors [5]. The genetic disorders include familial hypercholesterolemia (FH), hyperalphalipoproteinemia, and familial combined hyperlipidemia. Dyslipidemias arising from acquired factors often develop in the context of metabolic syndrome, including obesity, T2D and a sedentary lifestyle [5]. The prevalence of hypertriglyceridemia continues to rise and was estimated to affect about 35% in men and 21% in women [6].

Hypertriglyceridemia, increased remnant particles, small dense LDL (sdLDL) and low levels of high-density lipoprotein cholesterol (HDL-C) are the characteristics of highly atherogenic diabetic dyslipidemia (Fig. 1). Progression to moderate or severe hypertriglyceridemia may lead to multifactorial chylomicronemia syndrome.

The central pathogenetic causes of highly atherogenic diabetic dyslipidemia are abdominal obesity and insulin resistance. Visceral adipose tissue depots and subclinical inflammation promote increased release of free fatty acids and adipokines, which primarily reach the liver via the portal vein. The increased lipolytic activity of hormone-sensitive lipase (HSL) in peripheral insulin resistance plays a significant role here. The liver reacts to the increased supply of free fatty acids and hyperinsulinemia with increased lipogenesis, resulting in increased very low-density lipoprotein (VLDL) synthesis and fat storage (including liver steatosis). The hydrolysis of triglycerides (TG) in chylomicrons and VLDL by lipoprotein lipase (LPL) is impaired in insulin-resistant states. This impairment is attributable to two mechanisms. First, the overproduction of VLDL1 by the liver results in lipoproteins that are poor substrates for hydrolysis. Second, the LPL activity in muscle and adipose tissue is reduced due to insulin resistance and the proinflammatory adipokine milieu, including increased levels of interleukin-6 (IL6) and tumor necrosis factor (TNF). Although the chylomicrons and VLDL are unlikely to directly cause significant endothelial damage and the formation of atherosclerotic plaques because of their size, they hydrolyzed into relatively cholesterol-rich residual particles (remnants). In individuals without insulin resistance, this accumulation of remnants are normally rapidly metabolised, but in patients with insulin resistance, this degradation is slowed [7, 8].

Another consequence of insulin resistance and hypertriglyceridemia is the increased formation of sdLDL. With an increased concentration of VLDL and chylomicrons, their TG are increasingly transferred to HDL and LDL by the cholesteryl ester transfer protein (CETP) in exchange for cholesteryl esters. The decisive factor here is the concentration gradient of the TG. The LDL-bound TG are cleaved by the hepatic lipase, which is increased in the case of insulin resistance. This leaves sdLDL. SdLDL can easily penetrate the endothelium and have a longer half-life than normal LDL, properties that may explain their increased atherogenicity.

The decrease in HDL-C in hypertriglyceridemia and diabetic dyslipidemia develops similarly to sdLDL formation. Since activities CETP and hepatic lipase are increased in insulin resistance or T2D, exchange of TG from the TG-rich lipoproteins (TRL) for cholesteryl esters on large HDL2 is increased. These TG are in turn hydrolyzed there by hepatic lipase, and smaller HDL3 particles (sdHDL) are formed, which are rapidly metabolized and are no longer available for reverse cholesterol transport and endothelial protection. Apoprotein A-I can then dissociate from the small HDL, it is filtered renally and degraded in the renal tubular cells. As a result, HDL-C in plasma is low in patients with diabetic dyslipidemia. In addition, reduced activity of ABCA1 and reduced transfer of surface components of chylomicrons and VLDL to HDL contribute to the reduction in HDL-C.

## Global guidelines on lipid management to reduce cardiovascular risk

Various international and local health organisations have established comprehensive guidelines for lipid management in diabetes, focusing on LDL-C and non-high-density lipoprotein cholesterol (non-HDL-C) targets to mitigate CV risk. While specific recommendations vary, they collectively underscore the need for individualised treatment approaches based on overall CV risk, diabetes duration, and the presence of complications.

### American diabetes association (ADA) 2025 guidelines

The American Diabetes Association (ADA) 2025 guidelines underscore the importance of primary prevention through statin therapy for individuals aged 40 and older with diabetes [9]. For those younger than 40, statin use is advised if additional other CV risk factors are present. For individuals with diabetes and established ASCVD, high-intensity statin therapy is recommended to achieve an LDL-C reduction of ≥ 50% and a target of < 1.4 mmol/l (< 55 mg/dL), with ezetimibe or a PCSK9 inhibitor added if goals are not met. The ADA promotes shared decision-making to tailor treatment plans to individual patient profiles.

### American association of clinical endocrinology (AACE) guidelines

The American Association of Clinical Endocrinology (AACE) categorises individuals with T2D into three ASCVD risk categories: high, very high, and extreme [10]. For those at extreme risk, the guidelines recommend achieving an LDL-C < 1.4 mmol/l (< 55 mg/dL), a non-HDL-C level < 2.07 mmol/L (80 mg/dL) and an apoB level < 0.7 g/L (70 mg/dL). Individuals at very high risk, including those with diabetes or stage 3 or 4 chronic kidney disease and additional risk factors, are advised to target LDL-C levels < 1.81 mmol/L (70 mg/dL), non-HDL-C < 2.59 mmol/L (100 mg/dL), and apoB < 0.8 g/L (80 mg/dL).

### European society of cardiology (ESC) 2023 guidelines and focused update 2025

The 2023 European Society of Cardiology (ESC) guidelines on diabetes and ASCVD, together with the 2025 ESC focused update on lipid management, classify individuals with diabetes into moderate, high, very high, and, more recently, extreme cardiovascular risk categories. This stratification incorporates multiple determinants, such as age, duration of diabetes, target organ damage, other ASCVD risk factors, and time after events [11, 12]. For those at very high risk, individuals with existing ASCVD or severe target organ damage, as well as those with a projected 10-year CVD risk of ≥ 20% using the SCORE2-Diabetes model [13]. The guidelines advocate for an LDL-C target of < 1.42 mmol/L (55 mg/dL) and a non-HDL-C target of < 2.20 mmol/L (85 mg/dL).

## Cardiovascular risk stratification in diabetes

In the light of global guidelines, effective risk assessment allows for timely and targeted lipid management by identifying high-risk individuals who might benefit most from intensive therapy. Although diabetes is generally considered a high-risk condition for ASCVD, a thorough risk assessment should incorporate both conventional and diabetes-specific risk factors to ensure a more comprehensive evaluation (Table 1). In addition, results from various CV imaging approaches have been extensively evaluated. There is a broad consensus that an increased coronary artery calcium (CAC) Score (> 300) is associated with increased risk for myocardial infarction. However, a low CAC Score does not exclude coronary heart disease in symptomatic patients. In such cases, a computed tomography (CT) angiography is recommended for exclusion of relevant coronary stenoses. Moreover, there is a common interest in the early identification of patients with vulnerable plaques, as this may justify the timely initiation or intensification of intensive lipid lowering therapy [14]. For example, low attenuation plaques identified by CT angiography are associated with increased CV risk and should be considered as imaging-derived risk modifiers. These features complement established clinical and biochemical risk modifiers, including family history of premature CVD (men: <55 years; women: <60 years), elevated C-reactive protein (CRP), increased lipoprotein(a) Lp(a), metabolic dysfunction-associated steatohepatitis (MASH), HIV infection, and chronic immune-mediated inflammatory disorders. Additional modifiers include acute infection with respiratory viral infections, (e.g., influenza, and respiratory syncytial [RSV]) high-risk ethnicity (e.g. Southern Asian ancestry) psychological stress and psychosocial burden, social deprivation and adverse environmental exposures, such as air pollution, noise, chemical contaminates and broader effects related to climate change [15].

**Table 1:** .

Medical history	• Smoking status? • Known cAVD/CHD/PAD (cerebrovascular disease, coronary heart disease, peripheral artery disease)? • Premature CHD in first-degree relatives? • Age at diagnosis of diabetes? • Known nephropathy? • Known retinopathy? • Known neuropathy?
Laboratory tests	• Total cholesterol • Non-HDL cholesterol • HDL cholesterol • LDL cholesterol • Triglycerides • Lipoprotein(a) (Lp(a)) • GFR (glomerular filtration rate) • Creatinine • Albumin-to-creatinine ratio (UACR) in a spot urine sample • HbA1c
Imaging and further diagnostic tests	• Office/clinic blood pressure measurement • If necessary, 24-hour ambulatory blood pressure monitoring • Carotid Doppler • If necessary, abdominal-leg vascular Doppler • If necessary, ankle-brachial index (ABI)

According to the ESC 2023 guidelines, individuals with pre-existing ASCVD or severe end-organ damage are automatically classified as very high-risk, independent of other risk factors [12]. For patients aged 40 to 69 years with T2D but without ASCVD or severe end-organ damage, the SCORE2-Diabetes model offers the first validated risk score tailored for this population. It integrates conventional and diabetes-specific factors, such as age at diagnosis, hemoglobin A1c (HbA1c), and estimated glomerular filtration rate (eGFR), into the SCORE2 algorithms and accounts for four distinct European CV risk regions [5]. By combining these factors, SCORE2-Diabetes provides a 10-year prediction of CV events, enabling a more nuanced risk stratification compared to traditional tools.

The importance of structured risk assessment is underscored by findings from the SANTORINI study, which demonstrated that clinical judgment often underestimates CV risk. In this large European study, only 70.8% of patients were classified by physicians as very high-risk, whereas objective reassessment placed 91.0% of patients in this category [16]. This underestimation highlights the limitations of relying solely on clinician experience and the need for standardised, evidence-based risk assessment frameworks to guide treatment intensity.

## Lipid treatment targets in diabetes

Lipid abnormalities in diabetes extend beyond elevated LDL-C and include mixed dyslipidemia, particularly in patients with T2D. Mixed dyslipidemia is characterised by elevated TG and small, dense LDL particles, making LDL-C alone an insufficient marker of atherogenic risk [17]. The prevalence of mixed dyslipidemia in T2D ranges from 37% to over 90% depending on glycemic control, age, gender, and ethnicity [8, 18–20]. In type 1 diabetes (T1D), lipid abnormalities are less well characterised but frequently include low HDL-C and high LDL-C [7]. Notably, TG levels in well-controlled T1D are often normal or even slightly reduced, with one study showing 49% lower TG in men and 31% lower in women compared to healthy controls [21]. However, severe hypertriglyceridemia (TG > 11.3 mmol/L [1000 mg/dL]) can occur in the setting of diabetic ketoacidosis, posing a risk for acute pancreatitis.

Furthermore, the LDL-C estimation methods, including Friedewald formula, may not be reliable in patients with TG ≥ 400 mg/dL (4.5 mmol/L), or with TG ≥ 150 mg/dL (1.69 mmol/L) and LDL-C < 70 mg/dL (1.8 mmol/L) [22, 23]. Novel LCL-C calculation formulas, Martin formula and Sampson formula, may improve accuracy, particularly for high-risk populations [24].

LDL-C alone does not fully reflect atherogenic risk, particularly in mixed dyslipidemia, where TRLs (VLDL and remnants) contribute significantly to atherosclerosis. These lipoproteins contain cholesterol (remnant cholesterol), which is strongly associated with ASCVD [25]. Since LDL-C measurement excludes these particles, it underestimates the total atherogenic lipoprotein burden in these patient populations.

## Non-HDL-cholesterol as a marker targeting all atherogenic lipoproteins

To better capture risk, non-HDL-C is recommended as a secondary treatment target [2, 9]. Non-HDL-C calculated as total cholesterol minus HDL-C, provides a simple and rapid measure that can be obtained without additional laboratory measurements and in the non-fasting state without compromising accuracy. Unlike TG, it is minimally affected by intra-individual variability.

Non-HDL-C in the fasting state accounts for cholesterol in all apoB-100-containing lipoproteins, including VLDL, IDL, LDL, and Lp(a), providing a more comprehensive marker of ASCVD risk [20]. Given the causal role of Lp(a) in ASCVD, the measurement of Lp(a) is recommended in individuals at CV risk to identify those who may benefit from intensified management of modifiable risk factors and potential future Lp(a)-lowering strategies [26]. In the 2022 consensus statement by the European Atherosclerosis Society (EAS), Lp(a) levels above 125 nmol/L (50 mg/dL) are considered indicative of high CV risk [27].

Alternatively, apoB measurement directly quantifies the number of atherogenic particles and correlates well with non-HDL-C levels. Although the composition of remnant particles may change independent from cholesterol content, increases in the concentration of remnant lipoproteins, rather than simply loading existing lipoproteins with more TG, non-HDL-C and apoB are superior to TG alone in assessing risk [25, 28]. Additionally, studies have suggested that apoB outperforms LDL-C in the prediction of myocardial infarction events [29, 30].

Non-HDL-C targets are generally set 0.78 mmol/L (30 mg/dL) higher than LDL-C targets and should prompt similar treatment intensification if not achieved. LDL-C-lowering therapies also reduce non-HDL-C levels, because remnant cholesterol carrying apoB-100 lipoprotein particles are also removed from circulation by the LDL receptor in the liver [31, 32]. Despite clear treatment guidelines, lipid target attainment in individuals with diabetes remains suboptimal, particularly among those at high or very high CV risk. Data from a German-Austrian registry (2020–2022) showed low use of lipid-lowering therapies, especially in T1D (statins: 19.3%, ezetimibe: 2.2%, PCSK9 inhibitors: <1%), with only 6.2% of very high-risk and 11.0% of high-risk individuals reaching LDL-C targets [33]. In T2D, statin use was higher (45.7%), yet LDL-C goals were still met by just 11.8% (very high risk) and 16.3% (high risk), with similarly low non-HDL-C attainment. These findings align with broader data from the EUROASPIRE V survey, which assessed the implementation of European lipid management guidelines in coronary patients. In this cohort, 39.6% of individuals with self-reported diabetes achieved LDL-C < 1.8 mmol/L (70 mg/dL), while 38.6% reached non-HDL-C < 2.6 mmol/L (100 mg/dL) [34].

The data highlight persistent gaps in lipid management, indicating that recommended LDL-C and non-HDL-C targets are frequently unmet in individuals with diabetes. Given the high CV risk in this population, optimising lipid-lowering strategies in clinical practice is essential. This underscores the need for a structured approach to treatment, including the selection of appropriate lipid-lowering therapies and combination regimens to enhance goal attainment and reduce ASCVD risk.

## Lifestyle modifications and plasma lipids

For individuals with fasting TG ≥ 1.7 mmol/L (150 mg/dL) or non-fasting TG ≥ 2.0 mmol/L (175 mg/dL), lifestyle interventions are a key component of TG management. The 2021 American College of Cardiology Expert Consensus Decision Pathway on the Management of ASCVD Risk Reduction in Patients with Persistent Hypertriglyceridemia recommends a comprehensive approach, including assessing secondary causes and lifestyle habits such as diet, alcohol intake, and physical activity [35]. A heart-healthy dietary pattern should be emphasised, focusing on reducing added sugars, refined carbohydrates, and saturated fats while increasing fibre, healthy fats, and omega-3 fatty acids. Alcohol should be restricted or completely eliminated, particularly for individuals with TG ≥ 5.6 mmol/L (500 mg/dL). Regular aerobic activity, at least 150 min of moderate-intensity or 75 min of vigorous-intensity exercise per week, is recommended, alongside a weight loss target of 5–10% for individuals living with overweight or obesity. More stringent dietary modifications, including stricter fat and sugar restrictions, are advised for those with TG ≥ 11.3 mmol/L (1000 mg/dL) due to the heightened risk of pancreatitis. Referral to a dietitian or nutritionist or other supportive services may be beneficial, and continuous monitoring is necessary to assess and adjust interventions as needed. These measures can significantly improve triglyceride levels and overall lipid profiles.

## Lipid-lowering drugs in diabetic dyslipidemia

While achieving LDL-C and non-HDL-C targets remains the primary focus, real-world data suggest that many patients fail to reach these thresholds with statins alone. This highlights the need for adjunct therapies and emerging lipid-lowering strategies.

The following section explores current and upcoming treatment options and evidence-based strategies for lipid management in diabetes.

### Statins

Statins inhibit HMG-CoA reductase, the enzyme critical for cholesterol synthesis in the liver. This leads to an upregulation of LDL receptors, enhancing LDL-C clearance from the bloodstream. Statins are highly effective at reducing LDL-C and have consistently demonstrated reductions in cardiovascular morbidity and mortality in diabetes. Depending on the intensity of statin therapy, LDL-C reductions of at least 50% can be achieved with atorvastatin (≥ 40 mg daily) or rosuvastatin (≥ 10 mg daily). However, if the LDL-C baseline level suggests that monotherapy may be insufficient to reach the target, combination therapy with ezetimibe should be initiated promptly [36].

While generally safe and well-tolerated, statins may cause muscle-related symptoms in a minority of patients. A meta-analysis of 19 randomised controlled trials indicated a slight increase in muscle complaints during the first year of treatment, though only a fraction of these cases were directly attributable to statin use [37]. For patients who cannot tolerate statins or experience inadequate LDL-C reductions despite therapy, alternative or combination approaches should be considered. Although statins can modestly increase the risk of type 2 diabetes (proportional increased risk by 10–36% or increased incidence of 0,1–1,3% per year) [38], their overall benefits in reducing CV events outweigh this risk, making them indispensable for high-risk individuals [39].

### Ezetimibe

Ezetimibe inhibits intestinal cholesterol absorption and can reduce LDL-C levels by an additional 20–25% when combined with statins. The IMPROVE-IT trial demonstrated that ezetimibe, when added to simvastatin, reduced CV events by 6% [40]. Among patients with diabetes, who comprised 27% of the trial cohort, the relative risk reduction was even more pronounced at 14% over four years. These findings highlight the importance of ezetimibe as an adjunctive therapy, especially in high-risk individuals who fail to achieve LDL-C targets with statins alone.

### Bempedoic acid

Bempedoic acid is a prodrug activated in the liver, where it inhibits ATP-citrate lyase, a key enzyme in cholesterol synthesis. It lowers LDL-C by 18.1% when combined with statins and by 24.5% as monotherapy. The CLEAR Outcomes trial demonstrated a 13% reduction in CV events among statin-intolerant patients, with no significant increase in diabetes risk or negative effects on HbA1c [41, 42]. Bempedoic acid is well-tolerated, with only mild increases in uric acid and gout incidence reported. When combined with ezetimibe, LDL-C reductions of 36.2% can be achieved, providing an effective alternative for patients intolerant to statins or those requiring therapy intensification [43].

### PCSK9 inhibitors

PCSK9 inhibitors, including alirocumab and evolocumab, are monoclonal antibodies that bind to circulating PCSK9, preventing the degradation of LDL receptors and enhancing LDL-C clearance. These agents reduce LDL-C by 60% on top of statin therapy and have demonstrated significant CV risk reductions in people with diabetes [44, 45]. The ODYSSEY DM-DYSLIPIDEMIA trial showed that in individuals with T2D and mixed dyslipidemia on maximally tolerated statins, alirocumab significantly reduced non-HDL cholesterol by 32.5% compared to usual care, along with LDL-C, apoB, and LDL particle number [45]. The FOURIER and ODYSSEY OUTCOMES trials reported relative risk reductions in CV events of 17% and 16%, respectively [44, 46], with greater absolute benefits observed in people with diabetes [47, 48].

### Beyond monoclonal antibody PCSK9 inhibitors

Inclisiran, a small interfering RNA targeting PCSK9 administered every six months (two injections per year), offers sustained LDL-C reductions of approximately 50% [49] regardless of glycemic status or body-mass-index (BMI) [50]. The simplified dosing regimen may allow for optimal adherence, thereby facilitating the full therapeutic effect [51]. While long-term CV outcomes from the ORION 4 trial (NCT03705234) are pending, inclisiran represents a promising option for improving adherence and reducing residual risk.

### Cholesteryl ester transfer protein (CETP) inhibitors

CETP inhibitors, such as anacetrapib and obicetrapib, reduce the transfer of cholesteryl esters from HDL to TRLs, thereby lowering TGs and apo B-containing lipoproteins. While earlier CETP inhibitors failed to demonstrate CV benefits, anacetrapib showed promise in the REVEAL trial, reducing ASCVD events likely due to decreases in non-HDL-C. However, anacetrapib was found to accumulate in adipose tissue with prolonged use, raising safety concerns; therefore, its development was halted. Obicetrapib, the most potent CETP inhibitor to date, is currently under investigation to determine its CV efficacy. Obicetrapib has demonstrated promising lipid-lowering efficacy in recent clinical trials while being well tolerated. In the BROADWAY trial, which included 2,530 individuals with ASCVD and heterozygous familial hypercholesterolemia, obicetrapib led to a 33% LDL-C reduction at day 84 compared to placebo [52]. Similarly, the TANDEM trial, which evaluated a fixed-dose combination of obicetrapib and ezetimibe, reported a 48.6% LDL-C reduction compared to placebo [53]. Moreover, a meta-analysis on CETP inhibitors suggests a 16% reduction in the risk of new-onset diabetes and a trend toward improved glycemic parameters [54].

The potential CV benefits of obicetrapib’s lipid-lowering and metabolic effects are being evaluated in the PREVAIL CV outcomes trial (NCT05202509).

### Fibrates

Fibrates, which are peroxisome proliferator-activated receptor alpha (PPAR-α) agonists, have been used for decades to manage dyslipidemia. They enhance fatty acid oxidation, reduce hepatic triglyceride synthesis, and increase lipoprotein lipase activity, thereby lowering triglycerides and VLDL. While early studies, such as the VA-HIT trial, demonstrated CV benefits in patients with low HDL-C, later trials have been less conclusive. The FIELD trial and the ACCORD study found no significant reduction in CV events with fenofibrate as a primary endpoint [55, 56]. However, subgroup analyses suggested potential benefit in individuals with high baseline TG and low HDL-C, indicating that specific patient populations might derive some advantage. This lipid constellation was considered prospectively in the most recent trial, PROMINENT, evaluating pemafibrate in 10,497 individuals with T2D and mixed dyslipidemia at high CV risk [57]. Despite achieving significant reductions in VLDL cholesterol (−25.8%), remnant cholesterol (−25.6%), and apolipoprotein C3 (apoC3) (−27.6%), pemafibrate unexpectedly increased apoB (+ 4.8%) and did not reduce CV events (hazard ratio: 1.03; 95% CI, 0.91 to 1.15). The trial was terminated early for futility. Adverse effects included increased renal events and venous thromboembolism, though pemafibrate was associated with a lower incidence of nonalcoholic fatty liver disease.

Fenofibrate and pemafibrate both demonstrate substantial TG-lowering effects, with fenofibrate reducing TG by 30–50% and pemafibrate by ~ 26% in patients with diabetes [57, 58]. Therefore, they may be effective for preventing complications like pancreatitis in individuals with severe hypertriglyceridemia. However, their combination with statins should be approached cautiously due to potential adverse effects and until today there is no evidence for CV benefit in statin-treated patients.

### Omega-3 fatty acids

Omega-3 fatty acids, particularly eicosapentaenoic acid (EPA) and docosahexaenoic acid (DHA), lower TG by reducing hepatic very-low-density lipoprotein (VLDL) synthesis and enhancing lipoprotein clearance [59]. Approximately 3 to 4 g/d of EPA plus DHA are necessary to reduce hypertriglyceridemia by 20–50% [60] and may be considered for treatment of severe hypertriglyceridemia.

However, their role in CV prevention remains debated, as clinical outcomes have varied depending on the formulation and dose used. Mixed EPA/DHA formulations, such as those investigated in ASCEND and STRENGTH [61, 62], failed to demonstrate significant CV benefits, potentially due to the inclusion of DHA, differences in bioavailability, or suboptimal dosing.

In contrast, highly purified EPA, icosapent ethyl, demonstrated a 25% reduction in major CV events in the REDUCE-IT trial, despite only a 17% TG reduction [63]. However, concerns were raised regarding the use of mineral oil as a placebo, which may have increased inflammatory markers and biased the results in favor of EPA [64]. Even when adjusting for these potential confounders, modeling studies indicate that the CV benefits of EPA would not be entirely negated [65]. Supporting data from the EVAPORATE trial showed that EPA slowed coronary atherosclerosis progression [66], while the RESPECT EPA trial further reinforced its CV benefits [67].

In contrast, STRENGTH, using a mixed EPA/DHA formulation, found no benefit, suggesting DHA may counteract EPA’s protective effects [68]. Meta-regressions indicate a dose-dependent benefit for EPA, while DHA showed no clear advantage [61]. In vitro studies further support EPA’s anti-inflammatory role in stabilising cell membranes and reducing oxidative stress [69].

Given the discrepancy in outcomes between purified EPA and mixed EPA/DHA formulations, current data support the use of high-dose EPA (4 g/day) as an adjunct to statin therapy for high-risk individuals with TG between 1.5 and 5.6 mmol/L (135 and 499 mg/dL). However, further research is needed to fully elucidate the molecular mechanisms underlying EPA’s cardioprotective effects and clarify the role of DHA in CV health.

### Novel molecules reducing triglyceride-rich lipoproteins

Since triglyceride-rich lipoproteins are removed from the circulation by hepatic LDL-receptor, novel targets might reduce remnant cholesterol levels by addressing other targets involved in the metabolism of these lipoproteins, i.e. angiopoietin-related protein 3 (ANGPLT3) and apoC3. Patients with genetic defects in these targets have significantly lower blood levels of triglycerides, remnant cholesterol, and LDL-C. Therefore, first trials using corresponding therapeutic molecules lower these atherogenic cholesterol levels, and also even in patients with genetic defects in the LDL-receptor [70]. Therefore, new perspectives are evolving that combined hyperlipidemia might be treatable effectively in the near future and might even be suitable candidates for gene editing [71].

### RNA interference molecules

Plozarisan, an apoC3-targeting RNA interference molecule formerly known as ARO-APOC3, suppresses hepatic apoC3 synthesis, resulting in significantly lowering TG levels and non-HDL-C levels in patients with mixed hyperlipidemia [72, 73]. The phase 3 SHASTA-3 and SHASTA-4 trials will assess the safety and efficacy of plozarisan in reducing TG levels in patients with hypertriglyceridemia [74].

Zodasiran, an ANGPLT3-targeting RNA interference molecule formerly known as ARO-ANG3, is designed to selectively silence ANGPLT3 in the liver. In the phase 2b ARCHES-2 trial, zodasiran reduced TG levels up to 60%, accompanied by significant improvements across a broad range of atherogenic lipid parameters with a favorable safety and tolerability profile in patients with mixed hyperlipidemia [75]. In 2025, the phase 3 YOSEMITE trial was initiated to evaluate the efficacy and safety of zodasiran in patients with homozygous familial hypercholesterolemia [76]. With a potential applicability across severe and mixed dyslipidemia phenotypes, zodasiran targets multiple components of residual atherogenic lipid burden, thereby offering an integrated approach to reducing residual CV risk.

## Effects of SGLT-2 inhibitors and GLP-1 receptor agonists on plasma lipids and lipoproteins

Effective glucose management can reduce triglycerides and enhance lipid metabolism. This interplay between glycemic control and lipid metabolism suggests that some glucose-lowering therapies may influence lipid profiles, although it remains unclear whether these effects are direct or secondary to improvements in metabolic health.

Sodium-glucose co-transporter 2 (SGLT2) inhibitors, widely used for glucose-lowering in T2D, have demonstrated significant CV benefits. However, their impact on lipid profiles remains modest. A meta-analysis of 60 randomised trials including 147,130 individuals found that SGLT2 inhibitors slightly increased total cholesterol (+ 0.09 mmol/L), LDL-C (+ 0.08 mmol/L), and HDL-C (+ 0.06 mmol/L), while reducing triglycerides (−0.10 mmol/L) [77]. These effects were largely consistent across different SGLT2 inhibitors, with minor variations observed based on drug dose and ethnicity. Notably, Asian populations exhibited slightly greater HDL increases and TG reductions compared to non-Asian populations.

While the absolute changes in lipid parameters are modest, there appears to be a redistribution of cholesterol among different lipoprotein fractions, potentially reversing some of the adverse metabolic alterations associated with insulin resistance and diabetes. Mechanistically, SGLT2 inhibitors may enhance lipoprotein lipase activity, promote LDL conversion from VLDL, and induce metabolic shifts favoring lipid oxidation to generate energy [77]. Some studies suggest a reduction in sdLDL, a highly atherogenic subfraction, despite the overall rise in LDL-C. Additionally, the impact of SGLT2 inhibitors on apoB is not yet well defined, underscoring the need for further studies to elucidate the precise atherogenic effects of these agents [78]. Given the established CV benefits of SGLT2 inhibitors, these lipid effects likely represent a beneficial remodelling of lipid metabolism rather than a clinically significant concern and should not deter their use in high-risk patients.

GLP-1 receptor agonists (GLP-1RAs) primarily improve glycemic control and lead to weight loss in T2D but also modestly affect lipid metabolism. A meta-analysis of 76 trials found that semaglutide lowered LDL-C (−0.16 mmol/L) and total cholesterol (−0.48 mmol/L), while tirzepatide (−0.89 mmol/L) and ITCA 650 (−1.59 mmol/L) significantly reduced TG [79]. Moreover, tirzepatide demonstrated a dose-dependent reduction of apoC3 [80]. PEG-loxenatide was the only GLP-1RA to significantly increase HDL-Cl (+ 0.16 mmol/L). Therefore, GLP-1RAs may support CV health by favorably modulating lipid metabolism. However, their lipid-lowering effects are modest and should be viewed as an adjunct, not a replacement, for standard lipid therapy in high-risk patients.

## Conclusions

Lipid management remains a cornerstone of CV risk reduction in diabetes. Current guidelines prioritize LDL-C target attainment as the primary therapeutic goal, with non-HDL-C considered as secondary target. However, a comprehensive lipid assessment in individuals with T2D should extend beyond LDL-C to include triglycerides, non-HDL-C, Lp(a), apoB, and HDL-C, in order to improve CV risk stratification and enable more personalised treatment strategies.

Despite robust evidence supporting LDL-C and non-HDL-C lowering strategies, real-world treatment gaps persist, particularly in high-risk individuals. Moreover, guidelines emphasise a combination of statins, ezetimibe, PCSK9 inhibitors, and emerging therapies to optimise lipid control. While triglyceride-lowering therapies such as omega-3 fatty acids and fibrates reduce TG levels, their CV benefits remain uncertain. Future research should refine treatment strategies for mixed dyslipidemia, focusing on individualised lipid targets and precision medicine approaches.

## Data Availability

No datasets were generated or analysed in this study.

## Abbreviations

AACE: American Association of Clinical Endocrinology
ACCORD: Action to Control Cardiovascular Risk in Diabetes
ADA: American Diabetes Association
apoB: Apolipoprotein B
apoC3: Apolipoprotein C3
ASCVD: Atherosclerotic Cardiovascular Disease
BMI: Body–mass–index
CAC: Coronary artery calcium
CETP: Cholesteryl Ester Transfer Protein
CI: Confidence Interval
CT: Computed tomography
CV: Cardiovascular
CVD: Cardiovascular Disease
eGFR: Estimated Glomerular Filtration Rate
EPA: Eicosapentaenoic Acid
ESC: European Society of Cardiology
FIELD: Fenofibrate Intervention and Event Lowering in Diabetes
GLP-1RA: Glucagon–Like Peptide–1 Receptor Agonist
HbA1c: Hemoglobin A1c
HDL-C: High–Density Lipoprotein Cholesterol
HSL: Hormone–sensitive lipase
IL6: Interleukin–6
LDL-C: Low-Density Lipoprotein Cholesterol
Lp(a): Lipoprotein(a)
LPL: Lipoprotein Lipase
MASH: Metabolic dysfunction–associated steatohepatitis
NDR: National Diabetes Register
Non-HDL-C: Non–High–Density Lipoprotein Cholesterol
PCSK9: Proprotein Convertase Subtilisin/Kexin Type 9
PPAR: α–Peroxisome Proliferator–Activated Receptor Alpha
PROMINENT: Pemafibrate to Reduce Cardiovascular Outcomes by Reducing Triglycerides in Patients with Diabetes
sdLDL: small dense low–density lipoprotein
SGLT2: Sodium–Glucose Cotransporter 2
ST1RE: Steno Type 1 Risk Engine
T1D: Type 1 Diabetes
T2D: Type 2 Diabetes
TNF: Tumor necrosis factor
TG: Triglycerides
TRLs: Triglyceride–Rich Lipoproteins
VLDL: Very low–density lipoprotein
